# Obtaining World Coordinate Information of UAV in GNSS Denied Environments

**DOI:** 10.3390/s20082241

**Published:** 2020-04-15

**Authors:** Chengbin Chen, YaoYuan Tian, Liang Lin, SiFan Chen, HanWen Li, YuXin Wang, KaiXiong Su

**Affiliations:** 1 College of Physics and Information Engineering, Fuzhou University, Fuzhou 350108, China; 2 School of Economics and Management, Fuzhou University, Fuzhou 350108, China

**Keywords:** unmanned aerial vehicle (UAV), pan-tilt-based visual servoing (PBVS), track, navigation coordinate information, cloud computing

## Abstract

GNSS information is vulnerable to external interference and causes failure when unmanned aerial vehicles (UAVs) are in a fully autonomous flight in complex environments such as high-rise parks and dense forests. This paper presents a pan-tilt-based visual servoing (PBVS) method for obtaining world coordinate information. The system is equipped with an inertial measurement unit (IMU), an air pressure sensor, a magnetometer, and a pan-tilt-zoom (PTZ) camera. In this paper, we explain the physical model and the application method of the PBVS system, which can be briefly summarized as follows. We track the operation target with a UAV carrying a camera and output the information about the UAV’s position and the angle between the PTZ and the anchor point. In this way, we can obtain the current absolute position information of the UAV with its absolute altitude collected by the height sensing unit and absolute geographic coordinate information and altitude information of the tracked target. We set up an actual UAV experimental environment. To meet the calculation requirements, some sensor data will be sent to the cloud through the network. Through the field tests, it can be concluded that the systematic deviation of the overall solution is less than the error of GNSS sensor equipment, and it can provide navigation coordinate information for the UAV in complex environments. Compared with traditional visual navigation systems, our scheme has the advantage of obtaining absolute, continuous, accurate, and efficient navigation information at a short distance (within 15 m from the target). This system can be used in scenarios that require autonomous cruise, such as self-powered inspections of UAVs, patrols in parks, etc.

## 1. Introduction

Multi-rotor UAVs are widely used due to simple operation and convenient take-off and landing [1,2,3,4,5]. For example, the State Grid of China has now implemented unmanned aerial vehicle inspections of transmission lines above 110 KV, and its annual number exceeds 800,000 [1]. Traditional multi-rotor UAVs are controlled by artificial remote control, and the main disadvantages are high labor costs and limited cruising distance. With the development of UAV control technology, the multi-rotor UAV control method is gradually shifting to autonomous flight. The UAV autonomous flight environment is normally divided into cruising in the open environment according to the positioning point and channel cruising under the disturbed environment. As one of the most important components of autonomous flight, the autonomous navigation and positioning algorithm of multi-rotor UAVs has become a hotspot of current research. In addition, due to the limitations of the multi-rotor UAV structure, its payload and computing power are greatly restricted. Therefore, it is worthwhile to find a method that can realize autonomous and reliable positioning of the UAV with less resource occupation.

To obtain the specific position of the UAV, the most common method is receiving signals from GNSS, such as the GPS of the United States and the BeiDou Navigation Satellite System of China, and performing the calculation to obtain its current position, which can achieve meter-level positioning accuracy [6,7,8,9,10,11,12,13]. However, due to environmental factors, the GNSS positioning signal can be interrupted or deviate when the UAV approaches landmarks such as high-rise buildings and electrical towers [14,15,16], making the GNSS signal unable to provide stable and accurate positioning information for the UAV. The main causes of GNSS signal outage and degradation in flight include antenna obscuration, multipath, fading due to adverse geometry, and Doppler shift. Therefore, a new solution should be found to solve these problems.

In order to complete navigation under the circumstance where the GNSS signal is lost, many international strategies have been proposed, including GPS/INS integrated navigation [16,17,18], anti-interference design of GNSS equipment [19], and additional auxiliary sensors such as optical flow [20], radar [21,22], and other sensors for navigation assistance. With the development of machine vision systems in recent years, it has become possible to correct the flight path through machine vision [23,24,25,26,27,28,29,30,31,32,33,34]. With the vision system, UAV positioning navigation can completely get rid of the external positioning information, and stand-alone navigation can be achieved through the sensing equipment and camera that it carries. Now, the system has been verified in indoor environments [25], electricity systems [26], and agriculture [27]. At the same time, supplementary external navigation information can greatly improve the positioning accuracy of the UAV, thereby improving the robustness of the UAV flight [28,29]. The work in [20] presented a scheme to solve the problems of the low precision and weak stability of the micro inertial navigation system (MEMS) and introduced optical flow to fuse MEMS-IMU information with optical flow to improve further the robustness of UAV flight positioning control. The work in [25] proposed a new V-INS navigation strategy (vision aided inertial navigation systems), using a monocular camera to make UAVs operate in indoor environments without using GPS and to enable a micro-UAV to operate in a cluttered, unmapped, and gusty indoor environment. These methods can alleviate the loss of UAV positioning information to some extent, but during the long navigation process, the problem of data drift will occur due to the accumulation of errors. At the same time, with these methods, it is difficult to obtain the absolute geographical coordinate information of UAVs and it is hard to know the actual flight path of the UAV before the flight owing to the planned route being random. In the work in [28], a vision-based relative location (RL) method was proposed. Although it works well in an experimental environment, it is limited by finite perspectives and heavy calculations. The method of simultaneous localization and mapping (SLAM) for constructing geographic information for the system using real-time positioning and a map can provide location information during flight by generating an on-board environment map and share position estimates through communication networks to obtain relative locations [30]. However, it still cannot be applied to some environments in real life, such as open areas, flat corridors, indoor environments with sloping walls, and forests with leaves and branches. In many applications, it is not necessary to carry out complicated mapping work. Thus, mapping has become a complicated burdensome work in the system. In order to make the navigation information more accurate, many researchers have introduced absolute anchor points and compared the relative position information to eliminate errors and obtain more accurate UAVs. The current position, such as in [31], uses image analysis to estimate the current state of the UAV and then compares the relative position relationship between the landmark windmill and the UAV to obtain a more accurate UAV position; the work in [32] proposed a motion capture system based on a monocular, visual-inertial SLAM system that tightly fuses inertial measurements and observations of artificial visual landmarks, also known as “fiducials”, which constitute the map so that extended Kalman filtering can be used for tracking. This solution is now also commonly used in indoor UAV navigation. While the above schemes introduced reference landmarks, they need a large number of calculations. Moreover, many landmarks are needed for navigation considering that the landmark information can easily be lost. The work in [33] used landmarks as navigation targets during landing. The fixed camera was used to find the landmarks, and the relative position was obtained by pixel coordinates of the feature point. This method makes the landing error less than 0.11 m. However, due to the influence of flight and the poor processing capability of the image processing unit, the navigation speed is slow. The work in [34] used PTZ to track ground targets, transmitted image information back to the ground for calculation through image transmission equipment, and transmitted navigation information back to the aircraft for navigation. This scheme can perform high-speed high-altitude navigation, but errors are caused by the pixel coordinates of the feature point. Its navigation error is as high as five meters.

Overall, the coordination of multi-sensor devices is essential. At the same time, the introduction of landmarks can improve the positioning robustness of UAVs for a long time. However, in the current research, more equipment must be introduced for navigation during the operation process, which increases the complexity of the UAV itself and is not friendly for the UAV to track near targets. Therefore, in this work, we present a pan-tilt-based visual servoing system (PBVS), which uses a camera carried by a UAV to track the target, which is used as an anchor point to navigate during the UAV’s close-up operation. The main tasks of this system are listed in the following. The first is designing a recognition algorithm to recognize the landmark visually and calculate the deviation between the landmark and the optical axis. The landmarks recognized by this system are checkerboards, so all visual recognition calculations are carried out around checkerboard recognitions. The second is establishing a PTZ tracking system, which uses the fast-moving capabilities of the PTZ to perform field-of-view and coordinate target recognition. The algorithm tracks the target and stabilizes the camera, keeping the feature point of the target on the optical axis. The third is collecting real-time PTZ attitude information and calculating the relative information between the UAV and the target point through the geometric relationship. The fourth is calculating the absolute position information of the UAV according to the geographic location conversion algorithm, and this information is expressed by latitude and longitude. The major contribution of this work lies in the following. First, proposing an inertial compensation method for UAV flight vision foresight, it is used to capture and stabilize the navigation perspective in real-time without the need to calculate position information through the vision system, which greatly improves the calculation accuracy and reduces the system’s requirements for computing power. Second is constructing a new PTZ-visual servo control system that is used to obtain the world coordinate information of the UAV. Third is completing the auxiliary positioning and sensor fusion functions of the UAV through the cooperation of the UAV’s PTZ and the flight control system, it enables the UAVs to achieve fast positioning and navigation in the GNSS system interference environment. After the actual system verification, the solution can run stably on the platform we built, and its average positioning error is less than 0.87 m, which can provide good positioning information for the UAV.

## 2. The PBVS Model

In this paper, a method for obtaining navigation coordinates of a UAV, PBVS, is proposed. This method uses a UAV carrying cameras to carry out UAV navigation. In this section, the mathematical model of how the PBVS system obtains the navigation coordinates of the UAV is explained.

### 2.1. Problem Description

Currently, UAV autonomous cruise operation mode can be described as follows. First, the control center selects cruise routes and targets based on autonomous cruise requirements, then obtains the location and monitoring service time. The UAV cruise route is specified in this way. Then, according to the designated route, the UAV takes off from the tarmac and finds the target according to the cruise route, as well as takes pictures or videos and returns to the control center through wireless communication, and finally returns to the base. In the work process, the UAV has a fixed-point image shooting, such as power line insulator shooting. The characteristics of this type of operation are as follows.

1. The geographic location information of the operation target is known.
2. The target posture of each UAV operation remains unchanged.
3. The UAV with the camera is always tracking the target during the operation.

However, when the UAV collects images of the target in the autonomous cruise process, the GNSS signal is susceptible to interference such as power line inspection and building exploration, so other auxiliary sensing equipment such as visual navigation is needed. Current visual navigation methods allow navigation by carrying extra cameras. However, in practical work, due to the limited load capacity of the UAV, it is difficult to carry the equipment required for the operation. Methods for rebuilding UAVs at the lowest cost and navigating with the original camera carried by the UAV have become an emerging issue.

The UAV needs to use a single camera to judge the scene. In the process of UAV shooting, it is often necessary to obtain the shooting information in real-time rather than just one shot. At the same time, in the process of navigation, the camera needs to track the target in real-time to get the location information. In the process of tracking, we will face two challenges:

1. Ensuring that the target is not lost;
2. Ensuring the clarity of the target shooting.

First of all, to ensure that the target can be followed by the camera in real-time when the UAV has an irregular motion, the best way is to make the image appear in the center of the field of view, to ensure that the PTZ has sufficient response time for the camera movement in all directions [35,36]. Moreover, the unclear shooting of the tracking target is also an important reason for the loss of the target. Although many theories have been proposed to eliminate these influences, they are not comprehensive. There are two imaging methods for the camera: One is to take pictures with a global shutter, but if the exposure time is too long, the pictures will produce image paste phenomenon [37,38]. The other way is shooting with a rolling shutter, but if the progressive scanning speed is not enough, the shooting result may appear in any situation such as “tilt”, “swing uncertain”, or “partial exposure” [39,40]. The best way to overcome the blur is to reduce the relative motion between the camera and the target [37,38,39,40]. Therefore, the PBVS system keeps the target on the optical axis of the camera all the time through the pan-tilt control camera, to ensure the effectiveness of target tracking to the greatest extent.

### 2.2. Model Establishment

In this section, we will describe the model of UAV tracking and obtaining the coordinate, including the calculation of the UAV longitude and latitude, the establishment of the PTZ model, and the description of the camera imaging motion model.

Before establishing the model, the following assumptions can be made based on the actual situations of the UAV tracking the target object (anchor point):

1. In the autonomous flight of the UAV, it is necessary to confirm a specific target in advance. Therefore, it is assumed that the target can be identified by the UAV.
2. During the autonomous flight of the UAV, the targets inspected by the UAV are fixed, and the location is known in advance. Therefore, it is assumed that the three-dimensional world coordinates of the target object are known.
3. The UAV slowly approaches the target during the shooting process, so the angle of the UAV carrying the PTZ also changes slowly. When the computing power is sufficient, it is assumed that the camera carried by the UAV follows the anchor point in real-time, and the feature point of the anchor point always focuses on the camera’s optical axis.
4. The world geographic coordinate system and magnetic geographic coordinate system do not coincide, but the deviation is not large. Therefore, we can assume that the geographic and magnetic geographic coordinate systems are the same.
5. Although the Earth’s surface is spherical, the geographic coordinate projection of the UAV and the geographic coordinate projection of the anchor point are relatively close. It is assumed that the connection between the UAV projection and the anchor point projection is a straight line.
6. Although the Earth is not a regular sphere, the geographic coordinate projections of the UAV and the anchor point are relatively close. Therefore, it is assumed that the Earth is spherical and that the distance from any point on the spherical surface to the center of the sphere is unchanged, and its value is *R*. Based on these assumptions, the PBVS system model was established.

#### 2.2.1. Calculate Latitude and Longitude

**Figure 1:**
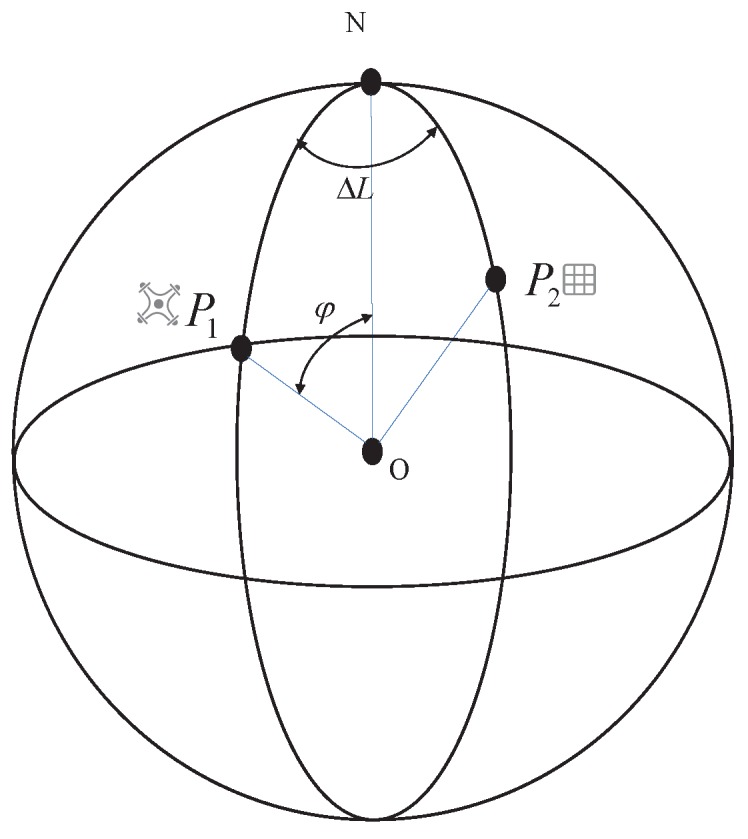
Schematic diagram of the PBVS model.

As shown in Figure 1, all points on the Earth can be represented by the geocentric geodetic coordinate system, where *O* is the center of the Earth, *N* is north, and any point on the Earth is recorded as $P_{n}\left(B_{n},L_{n},H_{n}\right)$, where $B_{n}$ is the latitude, $L_{n}$ is the longitude, $H_{n}$ is the elevation, and *n* is a subscript. It is assumed that the real-time coordinate of the UAV is $P_{1}\left(B_{1},L_{1},H_{1}\right)$, the coordinate of the target object is $P_{2}\left(B_{2},L_{2},H_{2}\right)$, its connection distance is *S*, and the relative azimuth between $P_{1}$ and $P_{2}$ is $A_{AZ}$.

The relationship between $P_{1}$ and $P_{2}$ satisfies Equation (1).

(1) $$\left[\begin{array}{c} B_{1}\\ L_{1}\\ H_{1} \end{array}\right]=\left[\begin{array}{c} 90-\varphi \\ L_{2}+\Delta L\\ H_{2}+\Delta H \end{array}\right]$$

where ΔL is the longitude difference between two points $P_{1}$ and $P_{2}$, which is represented as the dihedral angle between plane $NOP_{1}$ and plane $NOP_{2}$, φ is the complementary angle of $P_{1}$, which is shown as the connection angle between $P_{1}O$ and NO, and the height difference between point $P_{1}$ and $P_{2}$ is ΔH. We can get the current coordinates of $P_{1}$ with the known coordinates of $P_{2}$ after we calculate the relative difference between the two points. According to the triangle cosine theorem and spherical sine theorem, the complementary angle φ and longitude difference ΔL can be described as:

(2) $$\varphi =\arccos\left(\cos\left(\alpha \right)\cos\left(\beta \right)+\sin\left(\alpha \right)\sin\left(\beta \right)\cos\left(A_{AZ}\right)\right)$$

(3) $$\Delta L=\arcsin\left(\frac{\sin\left(\beta \right)\sin\left(A_{AZ}\right)}{\sin\left(\varphi \right)}\right)$$

where α is the complementary angle of the latitude of $P_{2}$, $\alpha =90-B_{2}$, *O* and the angle between $P_{1}$, $P_{2}$ and the center of the Earth is β, which can be expressed approximately as: $\beta =\frac{s}{R}\times \frac{180}{\pi }$.

#### 2.2.2. Establishment of the Camera Model

**Figure 2:**
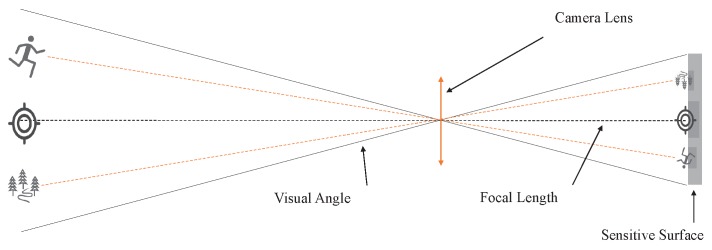
Pinhole camera model.

It can be known from Equations (1)–(3) that the coordinates of $P_{1}$ are related to $P_{2}$, *S*, $A_{AZ}$, *R*, and $H_{1}$. Assuming that the coordinates of $P_{2}$ are known and the distance *R* from any point on the Earth to the center of the Earth is known, $H_{1}$ can generally be measured by the built-in sensor. Therefore, to obtain the accurate value of $P_{1}$, the relative azimuth $A_{AZ}$ and the connection distance between $P_{1}$ and $P_{2}$ must be calculated. In the process of UAV work, the PBVS system can solve this problem. By Assumption 3, the feature points of the anchor point are always on the camera’s optical axis during the UAV flight shooting. The camera imaging follows the principle of pinhole imaging as shown in Figure 2. According to the pinhole imaging model, no matter what the camera shooting angle is, the object on the camera’s optical axis will always be in the center of the screen after imaging [41].

**Figure 3:**
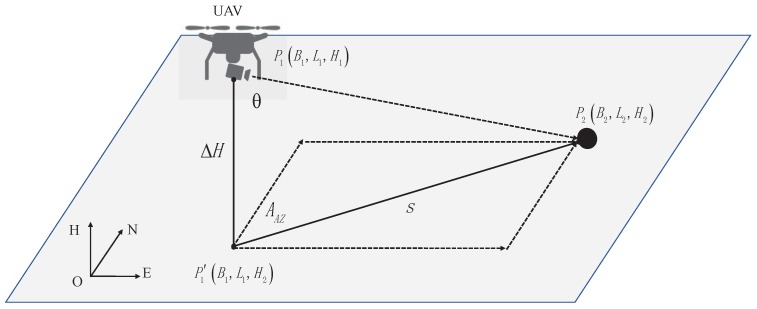
Schematic diagram of the spatial geometric relationship between the UAV and target object.

Because the optical axis is perpendicular to the camera imaging plane and the target is on the camera optical axis, the object at the center of the pixel field of view is perpendicular to the camera plane. It can be known from Assumption 3 that when the UAV approaches the target object, the shooting camera carried by the UAV is controlled by the stabilization PTZ to track the target object and can change its location according to the different positions of the UAV. Therefore, the spatial geometric relationship between the UAV camera and the target object is shown as in Figure 3.

**Figure 4:**
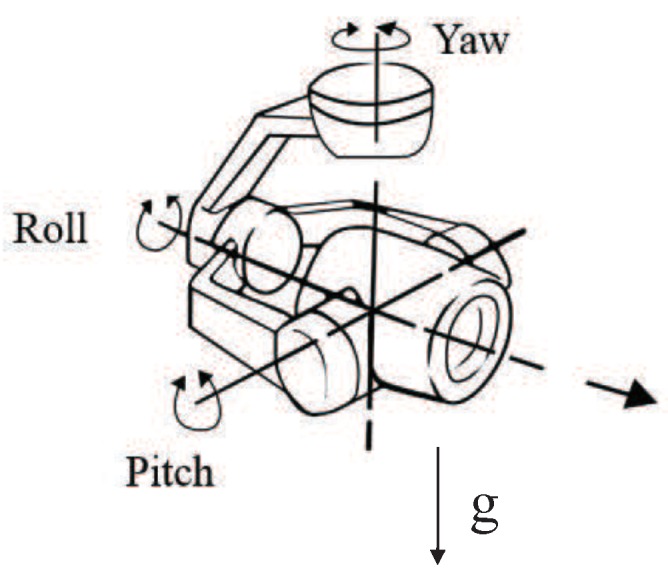
UAV PTZ-camera schematic.

For the convenience of calculation, we set up the navigation coordinate system as $\Sigma_{s}\left(X^{s},Y^{s},Z^{s}\right)$. Establish the UAV body coordinate system $\Sigma_{b}\left(X^{b},Y^{b},Z^{b}\right)$: the origin is the UAV mass center; the $X^{b}$ axis points to the UAV longitudinal axis (the head direction is positive); the $Y^{b}$ axis points to the UAV horizontal axis, and the right side is positive; the $Z^{b}$ axis is determined according to the right-hand rule; the rotation matrix from the UAV body coordinate system to the navigation coordinate system ${}_{b}^{s}R\in R^{3\times 3}$ is determined by the UAV attitude represented by Euler angle (Pitch,Roll,Yaw)(the rotation matrix from the navigation coordinate system to the UAV body coordinate system ${}_{s}^{b}R$ is known, ${}_{s}^{b}R=C_{y}\left(Roll\right)C_{x}\left(Pitch\right)C_{z}\left(Yaw\right){=}_{b}^{s}R^{-1}$ ). The camera coordinate system $\Sigma_{c}\left(X^{c},Y^{c},Z^{c}\right)$: the origin is the optical center; the axis $Z^{c}$ is along the direction of the optical axis; and $X^{c}$ and $Y^{c}$ meet the right-hand rule. The rotation matrix from the camera coordinate system to the UAV body coordinate system ${}_{c}^{b}R\in R^{3\times 3}$ is determined by the camera horizontal rotation angle ψ, roll angle φ, and pitch angle θ. The rotation matrix from the UAV body coordinate system to the camera coordinate system ${}_{b}^{c}R$ is known, ${}_{b}^{c}R=C_{y}\left(\varphi \right)C_{x}\left(\theta \right)C_{z}\left(\psi \right)={}_{c}^{b}R^{-1}$, with $C_{x}\left(\sigma \right)=\left[\begin{array}{ccc} 1 & 0 & 0\\ 0 & \cos\left(\sigma \right) & -\sin\left(\sigma \right)\\ 0 & \sin\left(\sigma \right) & \cos\left(\sigma \right) \end{array}\right]$, $C_{y}\left(\sigma \right)=\left[\begin{array}{ccc} \cos\left(\sigma \right) & 0 & \sin\left(\sigma \right)\\ 0 & 1 & 0\\ -\sin\left(\sigma \right) & 0 & \cos\left(\sigma \right) \end{array}\right]$, and $C_{z}\left(\sigma \right)=\left[\begin{array}{ccc} \cos\left(\sigma \right) & -\sin\left(\sigma \right) & 0\\ \sin\left(\sigma \right) & \cos\left(\sigma \right) & 0\\ 0 & 0 & 1 \end{array}\right]$. In the UAV system, the attitude of the camera is controlled in real-time by a three-axis camera PTZ. The schematic diagram is shown in Figure 4.

On the Earth, the static gravity acceleration *g* is vertically downward. Suppose the initial acceleration of the camera and the influence of the Earth’s magnetic field can be measured when the PTZ is stationary; the measured values are respectively $G_{1}$ and $M_{1}$, and the measured magnetic declination angle is expressed as δ.

(4) $$G_{1}=\left[\begin{array}{c} a_{x1}\\ a_{y1}\\ a_{z1} \end{array}\right]=\left[\begin{array}{c} 0\\ 0\\ g \end{array}\right]$$

(5) $$M_{1}=\left[\begin{array}{c} m_{x1}\\ m_{y1}\\ m_{z1} \end{array}\right]=\left[\begin{array}{c} \cos\left(\delta \right)\\ 0\\ \sin\left(\delta \right) \end{array}\right]$$

Supposing the UAV approaches at a low speed and the PTZ has little angle change, the accelerometer is basically stable, implying that the camera is approximately stationary. After rotation, the accelerometer $G_{2}$ and the magnetometer $M_{2}$ are in the following (rotation order is yaw-pitch-roll for the camera coordinate system):

(6) $$G_{2}=\left[\begin{array}{c} a_{x2}\\ a_{y2}\\ a_{z2} \end{array}\right]={}_{b}^{c}RG_{1}={}_{b}^{c}R\left[\begin{array}{c} 0\\ 0\\ g \end{array}\right]$$

(7) $$M_{2}=\left[\begin{array}{c} m_{x2}\\ m_{y2}\\ m_{z2} \end{array}\right]={}_{b}^{c}RM_{1}={}_{b}^{c}R\left[\begin{array}{c} \cos\left(\delta \right)\\ 0\\ \sin\left(\delta \right) \end{array}\right]$$

It can be calculated from Equation (6) that the acceleration value $G_{2}$ measured by the camera in real-time is as shown in Equation (8):

(8) $$G_{2}=\left[\begin{array}{c} g\cdot \sin\left(\phi \right)\cos\left(\theta \right)\\ -g\cdot \sin\left(\theta \right)\\ g\cdot \cos\left(\phi \right)\cos\left(\theta \right) \end{array}\right]$$

From Equation (7) and Equation (8), the expressions of the three attitude angles of the PTZ are:

(9) $$\left[\begin{array}{c} \theta \\ \phi \\ \psi \end{array}\right]=\left[\begin{array}{c} -\arctan\left(\frac{a_{y2}}{a_{x2}\sin\left(\phi \right)+a_{z2}\cos\left(\phi \right)}\right)\\ \tan\left(\frac{a_{x2}}{a_{z2}}\right)\\ \arctan\left(\frac{m_{x2}\sin\left(\phi \right)\sin\left(\theta \right)+m_{z2}\cos\left(\phi \right)\sin\left(\theta \right)+m_{y2}\cos\left(\theta \right)}{m_{x2}\cos\left(\phi \right)-m_{z2}\sin\left(\phi \right)}\right) \end{array}\right]$$

During the operation, the camera will be stabilized and controlled by a stabilization PTZ. Therefore, in theory, its roll angle ϕ equals zero, and the real-time attitude information of the camera can be expressed by:

(10) $$\left[\begin{array}{c} \theta \\ \phi \\ \psi \end{array}\right]=\left[\begin{array}{c} -\arctan\left(\frac{a_{y2}}{a_{z2}}\right)\\ 0\\ \arctan\left(\frac{m_{z2}\sin\left(\theta \right)+m_{y2}\cos\left(\theta \right)}{m_{x2}}\right) \end{array}\right]$$

Through simple geometric relations, we can get $s=\Delta H\times \tan\left(\theta \right)$, $A_{AZ}=\psi$. Therefore, the real-time camera information can be measured by the IMU and magnetometer carried by the camera. Therefore, by calculating the measured values and bringing them into Equation (2) and Equation (3), we can infer the current position $P_{1}$ of the UAV in the world geographic coordinate system.

#### 2.2.3. Description of Imaging in Camera Motion

We set the position of the shooting target in the navigation coordinate system to be ${}^{s}p_{0}={\left[\begin{array}{ccc} {}^{s}x_{0} & {}^{s}y_{0} & {}^{s}z_{0} \end{array}\right]}^{T}$, the position of the UAV in the navigation coordinate system to be ${}^{s}p_{1}={\left[\begin{array}{ccc} {}^{s}x_{1} & {}^{s}y_{1} & {}^{s}z_{1} \end{array}\right]}^{T}$, the installation position of the camera in the UAV body coordinate system to be ${}^{b}p_{2}={\left[\begin{array}{ccc} {}^{b}x_{2} & {}^{b}y_{2} & {}^{b}z_{2} \end{array}\right]}^{T}$, and the position of the target in the camera coordinate system to be ${}^{c}p_{3}={\left[\begin{array}{ccc} {}^{c}x_{3} & {}^{c}y_{3} & {}^{c}z_{3} \end{array}\right]}^{T}$. Each vector relationship satisfies Equation (11).

(11) $${}^{c}p_{3}{=}_{b}^{c}R\cdot_{s}^{b}R\cdot \left({}^{s}p_{0}-{}^{s}p_{1}\right){-}_{b}^{c}R\cdot {}^{b}p_{2}$$

The imaging position of the target in the image plane $I={\left[\begin{array}{cc} u & v \end{array}\right]}^{T}$ can be approximated by the pinhole model:

(12) $${\left[\begin{array}{ccc} u & v & 1 \end{array}\right]}^{T}=\frac{1}{{}^{c}z_{3}}\cdot M\cdot {\left[\begin{array}{ccc} {}^{c}x_{3} & {}^{c}y_{3} & {}^{c}z_{3} \end{array}\right]}^{T}$$

The parameter matrix of the camera $M=\left[\begin{array}{ccc} a_{x} & 0 & u_{0}\\ 0 & a_{y} & v_{0}\\ 0 & 0 & 1 \end{array}\right]$; $a_{x}$ and $a_{y}$ are the focal lengths of the camera, and ${\left[\begin{array}{cc} u_{0} & v_{0} \end{array}\right]}^{T}$ is the central pixel value of the image. By substituting Equation (11) into Equation (12), we can get:

(13) $$\left[\begin{array}{c} u\\ v \end{array}\right]=\frac{1}{{}^{c}z_{3}}C\cdot M\cdot {(}_{b}^{c}R_{s}^{b}R\left({}^{s}p_{0}-{}^{s}p_{1}\right){-}_{b}^{c}R{}^{b}p_{2})$$

With $C=\left[\begin{array}{ccc} 1 & 0 & 0\\ 0 & 1 & 0 \end{array}\right]$, the relationship between the pixel imaging and UAV motion can be obtained by the derivation of Equation (13):

(14) $$\dot{I}=J_{v1}\cdot v_{1}+J_{w1}\cdot w_{1}+J_{v0}\cdot v_{0}+J_{wc}\cdot w_{c}$$

where $\dot{I}={\left[\begin{array}{cc} \dot{u} & \dot{v} \end{array}\right]}^{T}$ is the moving speed of the target point in the actual image, $v_{1}={\left[\begin{array}{ccc} v_{x} & v_{y} & v_{z} \end{array}\right]}^{T}$ is the linear speed of the UAV movement, $w_{1}={\left[\begin{array}{ccc} w_{x} & w_{y} & w_{z} \end{array}\right]}^{T}$ is the angular speed, $v_{0}={\left[\begin{array}{cc} {\dot{x}}_{0} & {\dot{y}}_{0} \end{array}\right]}^{T}$ is the moving speed of the target, and $w_{c}={\left[\begin{array}{cc} \dot{\psi } & \dot{\theta } \end{array}\right]}^{T}$ is the rotation speed of the PTZ. $J_{v1},J_{w1}\in R^{2\times 3}$ refer to the image Jacobian matrix caused by the UAV’s linear speed and angular speed, and $J_{v0},J_{\mathrm{wc}}\in R^{2\times 2}$ refer to the image Jacobian matrix caused by the target and the PTZ’s movement

(15) $$J_{v1}=\frac{1}{{}^{c}z_{3}}\cdot N_{1}\left(e_{u},e_{v}\right)\cdot_{b}^{c}R$$

(16) $$J_{w1}=N_{2}\left(e_{u},e_{v}\right)\cdot_{b}^{c}R-\frac{1}{{}^{c}z_{3}}\cdot N_{1}\left(e_{u},e_{v}\right)\cdot_{b}^{c}R\cdot S{(}^{b}x_{2}{,}^{b}y_{2}{,}^{b}z_{2})$$

(17) $$J_{v0}=-\frac{1}{{}^{c}z_{3}}\cdot N_{1}\cdot_{b}^{c}R\cdot_{s}^{b}R\cdot C^{T}$$

(18) $$J_{wc}=\left[\begin{array}{cc} -\frac{a_{x}^{2}+{e_{u}}^{2}}{a_{x}} & \frac{e_{u}e_{v}}{a_{y}}\\ -\frac{e_{u}e_{v}}{a_{x}} & \frac{a_{y}^{2}+{e_{v}}^{2}}{a_{y}} \end{array}\right]$$

where $N_{1}\left(e_{u},e_{v}\right)=\left[\begin{array}{ccc} -a_{x} & 0 & e_{u}\\ 0 & -a_{y} & e_{v} \end{array}\right]$, $\left[\begin{array}{c} e_{u}\\ e_{v} \end{array}\right]=\left[\begin{array}{c} u\\ v \end{array}\right]-\left[\begin{array}{c} u_{0}\\ v_{0} \end{array}\right]$, $N_{2}\left(e_{u},e_{v}\right)=\left[\begin{array}{ccc} \frac{e_{u}e_{v}}{a_{y}} & -\frac{a_{x}^{2}+{e_{u}}^{2}}{a_{x}} & \frac{a_{x}e_{v}}{a_{y}}\\ \frac{a_{y}^{2}+{e_{v}}^{2}}{a_{y}} & -\frac{e_{u}e_{v}}{a_{x}} & -\frac{a_{y}e_{u}}{a_{x}} \end{array}\right]$, $S{(}^{b}x_{2}{,}^{b}y_{2}{,}^{b}z_{2})$ is the antisymmetric matrix corresponding to the installation position vector of the camera in the UAV body coordinate system ${(}^{b}x_{2}{,}^{b}y_{2}{,}^{b}z_{2})$, and the target depth information ${}^{c}z_{3}=\frac{\Delta H}{\cos\theta }$, which is included by $J_{v1}$ and $J_{w1}$.

## 3. Test Method

In this paper, we proposed an innovative PBVS physical model and its test method, which can be used for navigation of UAVs in GNSS-denied environments. At the same time, the PBVS navigation system has its limitations. This method is specifically used for UAV navigation of close-range fixed targets where GNSS signals are unavailable, rather than navigation of long-range random target operations.

During the autonomous flight of the UAV, the GNSS signal was used in the first place to approach the shooting target, when PVBS navigation stepped in and the UAV entered in GNSS-denied environments. First, the system identified the operation target to distinguish whether the target had been found. After the target was found, the UAV tracked the target by rotating the camera with a controllable three-axis PTZ, calculated the real-time UAV position, and then, gave feedback to the UAV flight controller. Then, the UAV entered the operation area. When the DOP value of the GNSS signal was greater than 10, the PBVS receiver was used as the navigation signal. Finally, the UAV used the control navigation algorithm to complete the autonomous flight path cruise and returned to areas where the GNSS signal was available to provide navigation information. In this paper, due to the limited computing power of the visual computing platform, we only performed PBVS system verification at low speeds of the UAV.

In the following sections, the PBVS system is described, including the architecture of the system, the visual algorithm, and PTZ controllers.

### 3.1. System Description

**Figure 5:**
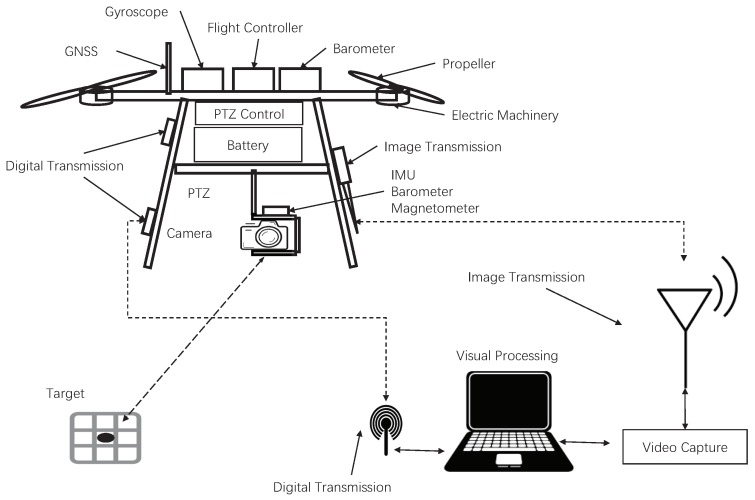
The experimental setup on multi-rotor UAVs.

In this system, we simulated the existing UAV system and independently built a UAV test platform. The experimental setup on our multi-rotor UAV is shown in Figure 5. It shows that it was not much different from ordinary aerial photography UAVs, which included a self-designed aircraft control system, four power units, aerial model power batteries, aerial PTZ, aerial cameras, and communication modules. In order to meet the test requirements, this system carried out a few necessary modifications, including attitude and azimuth measurement units (IMU, magnetometer, and altimeter) on the PTZ, used to detect the real-time attitude, orientation, and height information of the camera. The unit was rigidly connected with the camera and the RTK-GPS sensor for data comparison on the UAV, the image transmission unit, and the data transmission unit capable of data interaction with ground stations. Because the computing power of the onboard equipment was insufficient, a ground station was established for visual calculation processing of the UAV. Although this system brought a relatively large delay to the UAV system, this method performed image calculations more efficiently during low-speed flight, which verified the feasibility of PBVS navigation.

**Figure 6:**
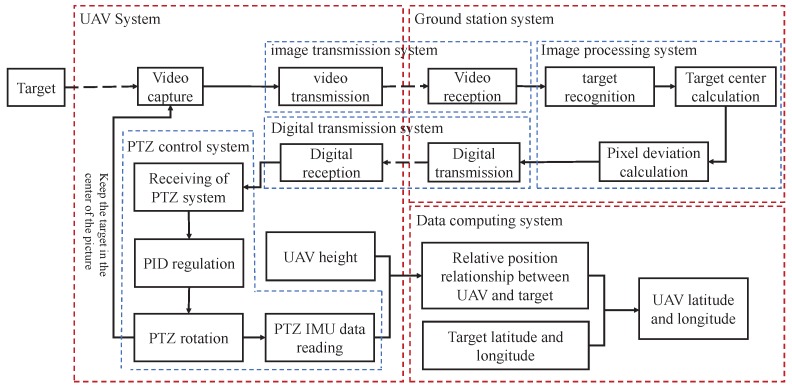
The architecture of the PBVS system for the multi-rotor UAVs.

The algorithm architecture of the system for the multi-rotor UAVs is shown in Figure 6. The purpose of this study was to measure the relative position information of the UAV and the landmark in real-time through the camera mounted on the UAV’s PTZ in real-time when the GNSS was inaccurate or invalid. The specific working principles are described as follows:

1. Image processing and target recognition: The camera mounted on the PTZ performed environmental video collection and transmitted the collected video back to the ground server for processing to identify whether the job target appeared in the image. When the target appeared, a pixel coordinate system was established by the picture pixels, and the coordinate value of the feature point of the target center in the pixel field of view was calculated. It was compared with the pixel coordinate value of the camera center point. The deviation value of the pixel center position from the target feature point position could be output and transmitted to the UAV PTZ control system.
2. PTZ control and target tracking: After receiving the deviation information between the pixel center position and the target feature point position, the UAV control system adjusted the angle of each axis of the three-axis PTZ with the input deviation value and moved the center of the camera’s field of view to the target feature point, that is to adjust the camera optical axis to the object. During the subsequent operations, the PTZ control system repeated this step in real-time to ensure that the target feature point was on the camera optical axis.
3. Position coordinate resolving: When the UAV’s PTZ locked the target at the center field of view, the PTZ’s current attitude and altitude information could be read by the PTZ’s sensors. Further, the UAV’s current latitude and longitude position could be calculated by the physical model derived in Section 2. The position information was input into the UAV control unit and navigation could be completed.

In the following sections, more detail about the visual system and the PTZ controllers is described.

### 3.2. Visual System

The vision system was composed of two parts: the target recognition algorithm and the target feature point identification. The main purpose of this system was to identify the work target and update the relative position of the work target. We needed to use different recognition methods in different scenarios because of the difference of target recognition, so in this design, the visual system was not the main content, and the visual recognition methods of different job targets were introduced in several documents [42,43]. Therefore, a simple method was introduced to verify the system. In this experiment, we used a calibration chessboard as the target object and regarded its center point as the feature point. The methods used in our experiments are described below.

**Figure 7:**
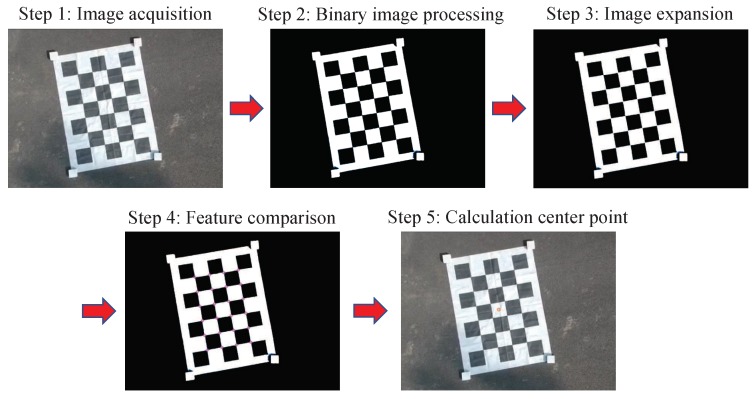
Method for identifying and obtaining the feature point coordinates.

In this system, it was necessary to control the optical axis of the camera to aim at the center of the target object in real-time. Therefore, the visual system must be used to identify the target and obtain its center point coordinates. The overall operation steps are shown in Figure 7.

1. Use the camera to collect marker images;
2. Binarize the images collected by the camera. The local average adaptive thresholding method was used to binarize the image, and the judgment threshold was obtained after equalization;
3. Dilate the image to separate the connections between the binarized black blocks, so as to obtain a clearer binarized image;
4. Conduct contrast detection of the target image, find the target pattern features in the image, and use the constraint conditions such as feature pattern aspect ratio, perimeter, and area to remove noise interference in the image;
5. Calculate the coordinates of the center position of the target.

The method of using vision to obtain the target object feature point is common and is not illustrated in this article. The purpose of this operation is mainly to process visually so that after the calculation of a complex target object, a simple mass point (the feature point) can be used to represent it, so as to provide convenience for the calculation of the spatial geometry relationship between the UAV and target object.

### 3.3. PTZ Control

In the PBVS system, a PTZ was used to drive the camera, so that the camera’s optical axis could always be aligned with the feature point of the target object to ensure that its imaging was near the center of the image plane with less distortion. The rotating platform had two independent tasks in the system:

- Stabilizing effect: The use of the PTZ could ensure that the vision acquisition system was not affected by the UAV’s under-drive control system. During fast and dynamic operation, the camera could overcome the jitter caused by the UAV flight, thus maintaining independence and enabling the vision system to work in a specific posture;
- Tracking: This significantly improved the visual servoing problem of the PBVS system, enabling the system to ensure that the camera could track the target object in real-time based on the feedback information provided by the vision system during the work process and keep the feature point of the target object always on the camera’s optical axis.

Due to the limitation of hardware computing power, the vision system had to be built on the ground to keep the video playing at a frequency of 15Hz, that is data must interact with the ground station through image transmission equipment and digital transmission equipment, which brought the PBVS system a 300 ms delay [44]. We used the UAV motion model to estimate the motion state for navigation estimation, so as to eliminate the control effect caused by delay [34,45]. Moreover, in the experiment, the visual servo problem was guaranteed by reducing the maximum flying speed of the UAV.

The three degrees of freedom (DOF) control of the PTZ were independent. When the PTZ θ was independently operated, the picture taken by the camera would move along the pixel coordinate system *Y*-axis; when the PTZ ψ was independently operated, the picture taken by the camera would be along the *X*-axis of the coordinate system; when the PTZ ϕ was operated independently, the image captured by the camera would be rotated along the optical axis of the camera. In the process of target tracking, only performing PTZ θ and ψ control took effect. Since the target was still and the UAV used the stabilized PTZ to keep the camera attitude stable, Equation (14) could be simplified as:

(19) $$\dot{I}=J_{v1}\cdot v_{1}+J_{wc}\cdot w_{c}$$

**Figure 8:**
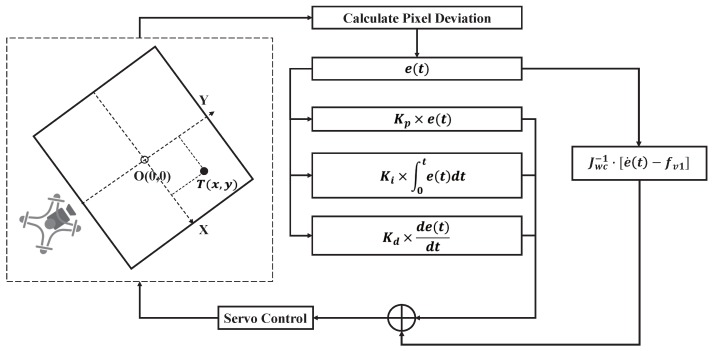
PTZ control system schematic diagram.

In the whole camera tracking control, it could be divided into motion compensation control and PID control. The specific control flow is shown in Figure 8.

System error signal:

(20) $$e\left(t\right)=\left[\begin{array}{c} e_{u}\left(t\right)\\ e_{v}\left(t\right) \end{array}\right]=\left[\begin{array}{c} u\left(t\right)-u_{0}\\ v\left(t\right)-v_{0} \end{array}\right]$$

The equation of motion of the target in the image plane can be obtained by deriving Equation (20):

(21) $$\dot{e}\left(t\right)=J_{v1}\cdot v_{1}+J_{wc}\cdot w_{c}$$

That is $\dot{e}\left(t\right)=f_{v1}+J_{wc}\cdot w_{c}$, where $f_{v1}=\frac{\cos\theta }{\Delta H}\cdot N_{1}\left(e_{u},e_{v}\right)\cdot_{b}^{c}R\cdot v_{1}$. Then, the motion compensation control signal is:

(22) $$w_{c}=J_{wc}^{-1}\cdot \left[\dot{e}\left(t\right)-f_{v1}\right]$$

In order to achieve faster response speed and a better control effect, we added PID control at the same time. The output of PID control was $w_{PID}={\left[\begin{array}{cc} {\dot{\psi }}_{PID} & {\dot{\theta }}_{PID} \end{array}\right]}^{T}$, and its expression is as Equation (23).

(23) $$w_{PID}=K_{p}\left[e\left(t\right)+\frac{1}{T_{I}}\int_{0}^{t}e\left(t\right)dt+T_{D}\frac{de(t)}{dt}\right]$$

where $K_{p}$ is the proportion coefficient, $T_{I}$ is the integral time constant, and $T_{D}$ is the differential time constant.

The output angular velocity of the whole system shall be $w=w_{c}+w_{PID}$.

## 4. Simulation and Experiment

In this section, we build a simulation platform for testing. Furthermore, we conduct real-world experiments to verify the proposed scheme.

The simulation environment was established very close to the real experiment. Besides, the simulation results were analyzed qualitatively and quantitatively, and the performance of autonomous navigation was evaluated. In the actual experiment, we built a real experimental platform to test the performance of the proposed system. We also tested the accuracy of autonomous navigation. Furthermore, we compared with other methods and proved the superiority of the PBVS method.

### 4.1. Simulation Experiment

#### 4.1.1. Simulation Environment

In the simulation environment, we could protect the UAV from wind and other external influences. At the same time, the UAV body vibration and sensor errors would not exist. In the simulation environment, we could process all kinds of data in real-time, including the collected image data. In the aspect of data reading, the UAV could read the flight trajectory without errors in the simulation environment. Furthermore, it could also control the experimental environment, including the adjustment of the landmark size, UAV size, UAV speed, and camera field of view. In the simulation environment this paper involved, we made the UAV fly without any interference, eliminating the transmission delay of the video, and enabling us to read the calculated PBVS data in real-time.

**Figure 9:**
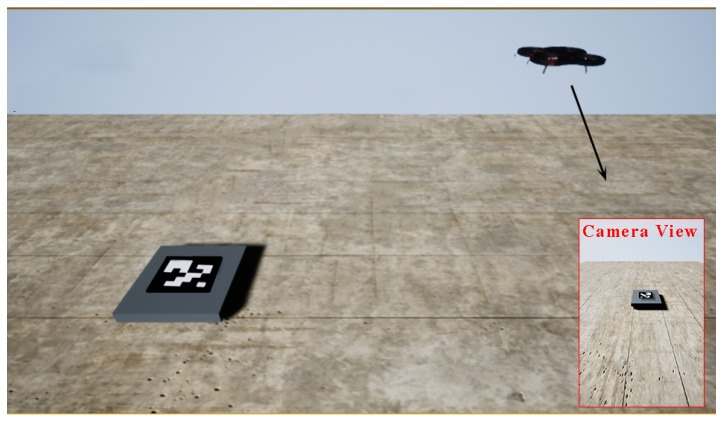
Simulation environment based on Microsoft AirSim.

We used the AirSim simulation platform based on the Unreal Engine 4 released by Microsoft to simulate the autonomous landing system. The platform provides the simulation environment, UAV model, application programming interface, and other resources close to the real-world environment. In this platform, we tested all kinds of possible situations, in which the loss of the GPS signal was simulated by no reading GPS data. First of all, we performed the autonomous flight on the AirSim platform and pasted the ArUco mark to increase its identifiability. Then, after taking off, the UAV would fly to any position about 15 m from the horizontal distance of the platform, to ensure that the image captured by the airborne camera (FOV is 50 degrees) could correctly identify the landing platform and then start to give the command of starting autonomous flight to the UAV. The whole simulation environment is shown in Figure 9.

#### 4.1.2. Simulation Results

**Figure 10:**
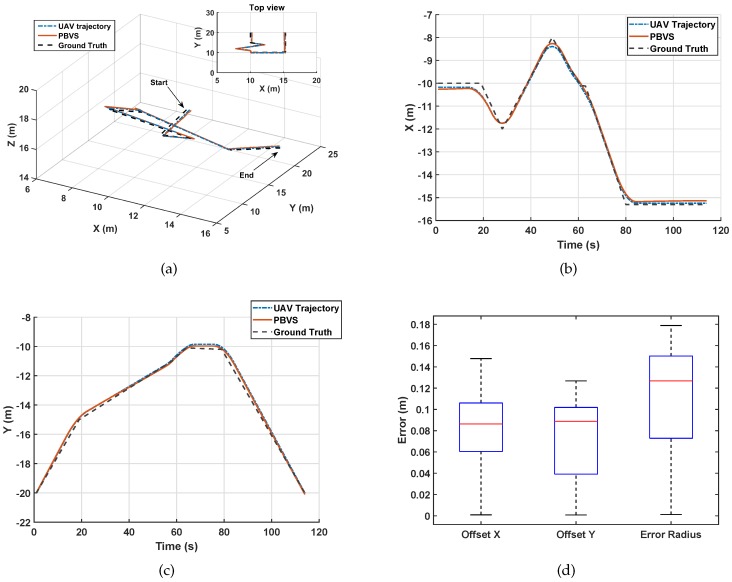
The flight simulation: (**a**) 3D trajectory of UAV; (**b**) the localization results in the X coordinate; (**c**) the localization results in the Y coordinate; (**d**) the trajectory error.

In the simulation platform, we designed the route for the UAV to take photos around and let the UAV use PBVS for navigation. According to Section 3, we first let the camera find the shooting target, obtain the location information, and then start the navigation. In the fusion control mode, we used the most classical Kalman filter to obtain more accurate position information. As shown in Figure 10a, we designed the UAV to fly in irregular routes.

In Figure 10a, blue represents the trajectory of the UAV, red the position information obtained by PBVS estimation, and black the target trajectory. From the figure, we can see that the UAV navigation line using PBVS was very close to the real preset path, and PBVS could effectively reflect the real path of the UAV.

As can be seen from Figure 10b,c, in the simulation environment, the PBVS basically coincided with the X-axis and Y-axis of the UAV trajectory. It could be seen from the simulation results that PBVS could accurately reflect the real position of the UAV in a relatively ideal environment, and it would not lose the accuracy of navigation due to the sudden change of UAV attitude. It can be seen from Figure 10d that the overall system error of PBVS in the simulation environment was small. It could be seen from the simulation that in the ideal environment, PBVS could obtain more accurate location information, and using PBVS could obtain a better navigation effect for the UAV.

### 4.2. Field Testing

#### 4.2.1. Experimental Platform Construction

**Figure 11:**
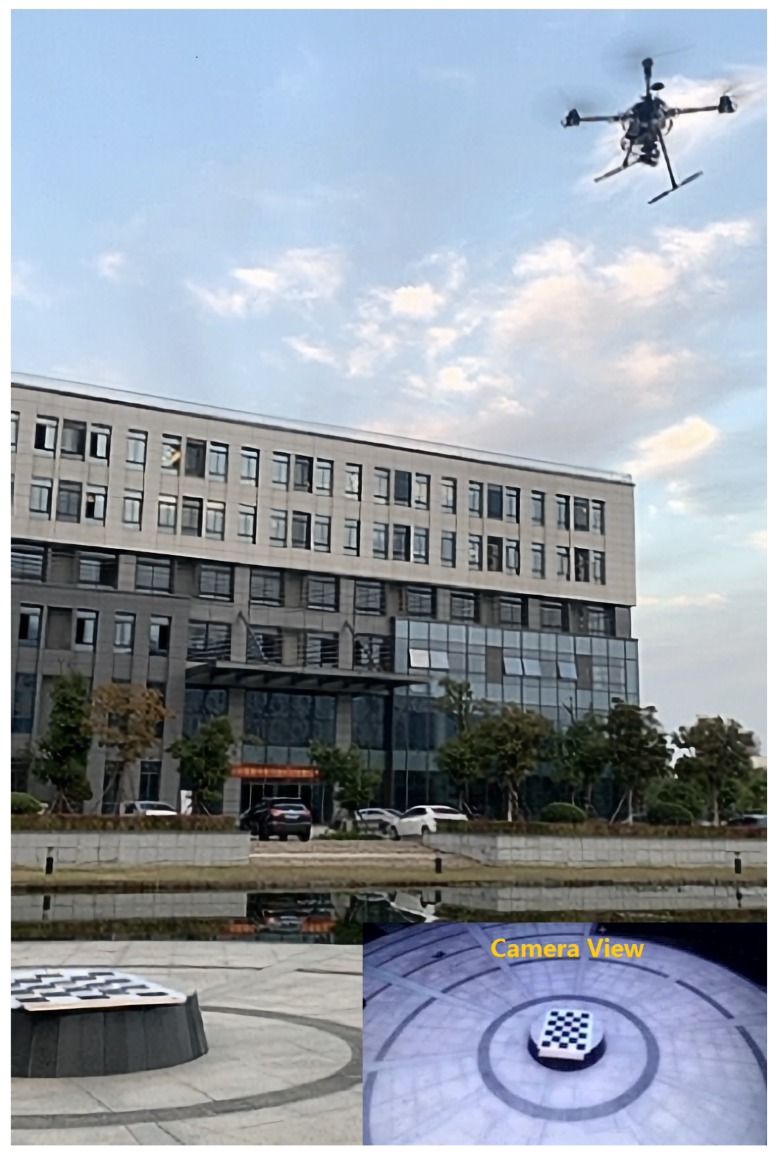
Outdoor experimental environment.

The test location of this paper was outside the Fuzhou University Science and Technology Park (the coordinates of the landmark are latitude 26.054807 and longitude 119.198058). The models and parameters of the experimental platform used in this paper are shown in Table 1. The outdoor experimental scenario is shown in Figure 11. The experimental platform was equipped with an RTK differential positioning module to provide high-precision positioning information for the UAV. This information was provided to the flight controller for reference only. The altitude information of the UAV in this paper was estimated by the on-board barometer. The barometer had temperature drift and other phenomena, and its reading drifted with time. Therefore, using the Kalman filter to process the altitude data obtained by the barometer could effectively suppress the drift of the aircraft altitude data [46,47,48].The aircraft was equipped with a PTZ controller. The tasks of the PTZ controller were as follows:

1. Receive and process the data sent by the ground station.
2. Read the camera and aircraft attitude data, RTK data, altitude data, etc., and return the current data to the ground station through the transmission module.
3. Control the rotation angle of the PTZ.

In the experiments of this paper, the reference RTK readout error was less than 10 cm, which was much smaller than the GPS error, so it was used as the UAV trajectory data of the UAV in this paper.

#### 4.2.2. System Response Speed Test

In this experiment, the calculation of the offset between the object feature point and the optical axis of the camera was performed at the ground station. Therefore, the image collected by the UAV should be transmitted back to the ground station through the image transmission device. The ground station ran the image processing algorithm. The calculated camera offset error was transmitted back to the PTZ controller, and the PTZ would take corresponding actions to adjust the camera attitude. Since the UAV was a dynamic system, the system delay had a great influence on the control effect. Assuming that the delay of the image transmission equipment could not be optimized, the image processing speed of the ground station determined the feasibility of the algorithm. The horizontal flight speed of the aircraft selected in this paper was 0.3 m/s. According to the image recognition algorithm proposed above, the video processing speed was calculated for 10 consecutive minutes. The fastest processing speed was 18.02 f/s, the slowest 12.54 f/s, and the average processing speed 15.36 f/s; most of the processing speed was concentrated between 13–16 f/s, which met the needs of subsequent experiments.

**Figure 12:**
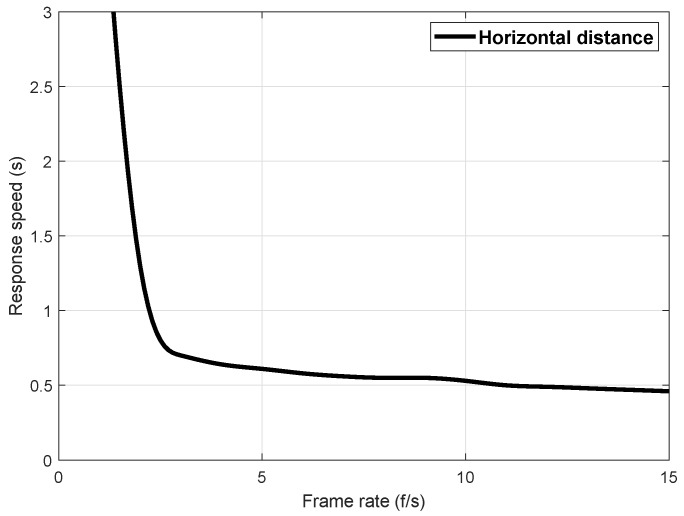
Image processing frame rate and PTZ response speed curve.

At the same time, we tested the speed and overshoot of the PTZ’s response based on image errors. Because the UAV had a dynamic system, its flight process was susceptible to the interference of environmental noise, which required the PTZ to adjust its angle as quickly and without overshoot as possible when it received the image offset error sent by the ground station. During the actual test of the system, the signal instability of the image transmission equipment may affect the frame rate of the ground station image processing. In addition, when the target landmark was far away or the inclination between the camera and the landmark reached a certain range, the recognition rate may decrease. In order to test the effect of the image processing frame rate on the response speed of the PTZ, the PTZ response speed test was artificially reduced to reduce the image processing frame rate. The results are shown in Figure 12. It can be seen that when the frame rate of image recognition was less than 5 f/s, the response speed of the PTZ was slow, which was not enough to meet the control requirements. When the image recognition frame rate was greater than 6 f/s, the response speed was basically stable at 0.5 s. At this time, with the increase of the image recognition frame rate, the response speed of the PTZ would not increase, and the delay of the response speed of the PTZ at this time was mainly determined by the transmission delay of the system and the response speed of the PTZ itself.

**Figure 13:**
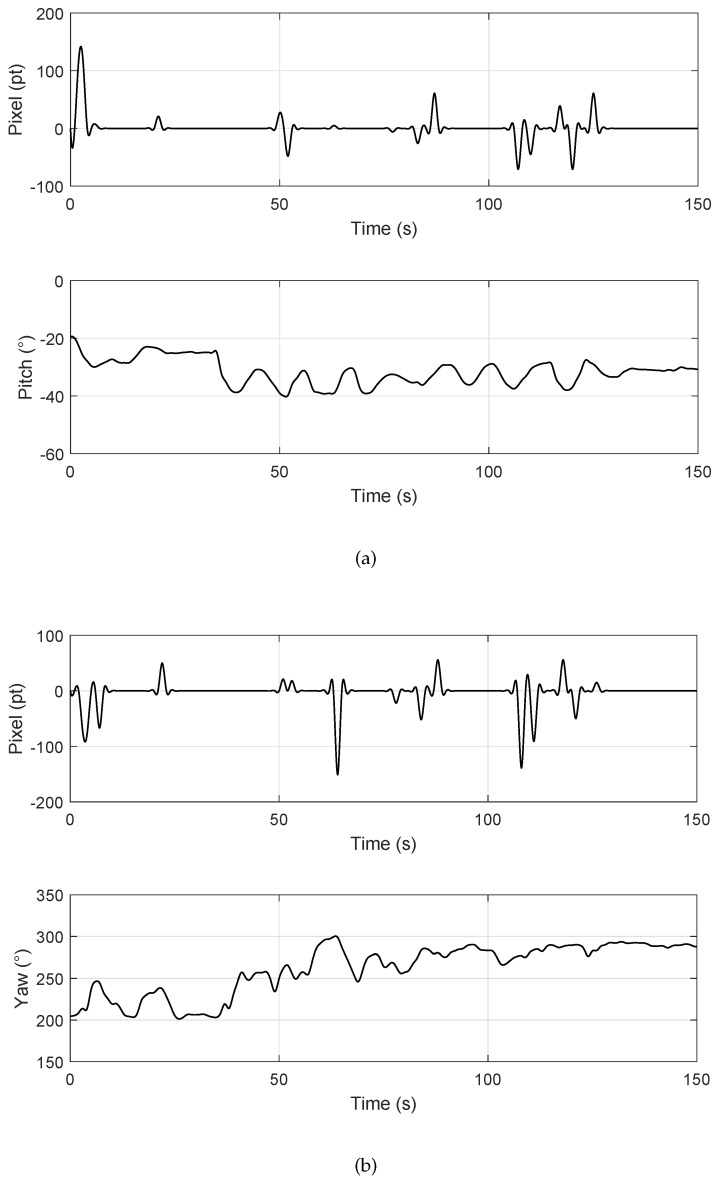
Rotation angles’ diagram: (**a**) Y-axis offset error and PTZ pitch angle; (**b**) X-axis offset error and PTZ yaw angle.

Figure 13 shows the relationship between the offset error of the camera image and the PTZ rotation angle. The PTZ could maintain the current rotation angle until its controller received the error signal, the PTZ. The UAV flew to a height of 3.5 m to test the targets. By controlling the UAV to make the artificial error, the PTZ acted accordingly after receiving the error. It could be seen that there was a certain degree of jitter in the PTZ angles, which was mainly caused by the vibration of UAV fuselage during the flight. The test results indicated that the average jitter of the PTZ pitch angle caused by the fuselage vibration was 2.1°, and the average jitter of the yaw angle was 3.3°. They had little effect on the control effect and the accuracy of the horizontal displacement estimation.

#### 4.2.3. UAV Flight Test

**Figure 14:**
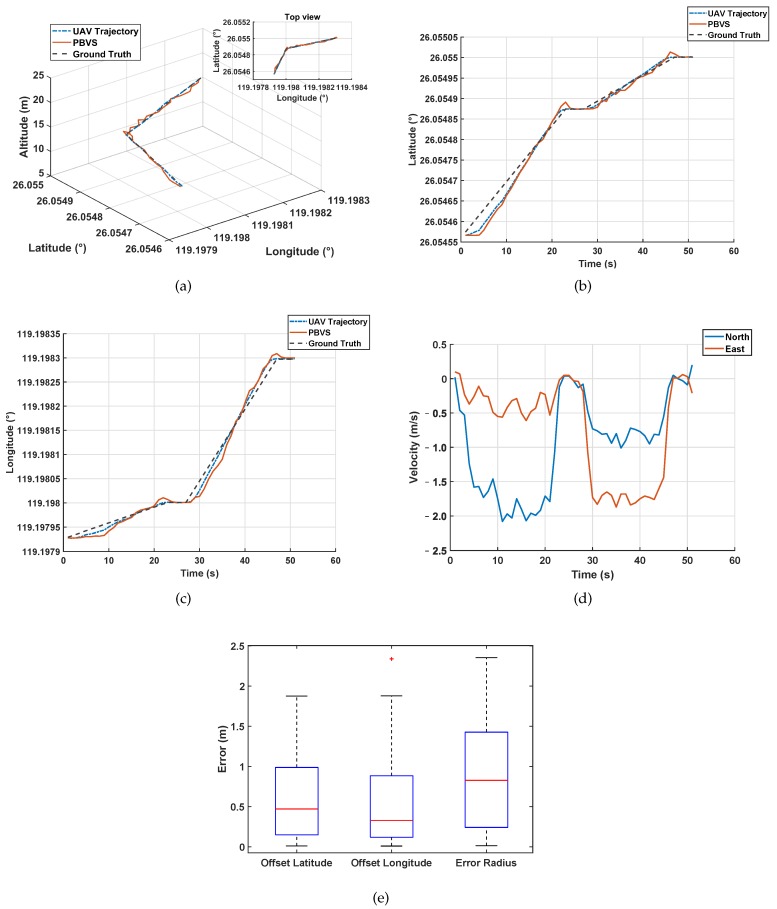
Actual UAV flight experiment: (**a**) flight three-dimensional diagram; (**b**) latitude diagram; (**c**) longitude diagram; (**d**) velocity diagram; and (**e**) error diagram.

In order to verify whether the UAV could use the estimated latitude and longitude for actual flight, an actual flight test was performed. The data read by the RTK and the estimated latitude and longitude data of the UAV around the object target (anchor point) were plotted as three-dimensional flight paths to verify the correctness of the flight trajectory, as shown in Figure 14.

The UAV first took off, hovered after takeoff, then flew to Point 1 at a maximum speed of 2 m/s, hovered, and changed direction to Point 2 and hovered next to it. In Figure 14a–c, the blue curve in the figure is the actual flight path measured by RTK, and the red curve is the UAV position data estimated from the PTZ rotation angle and its height. In an actual flight, the UAV would be affected by external factors such as wind and it will also be affected by the UAV system itself such as body shaking and processing delay. However, it can be seen that the red curve could roughly reflect the trajectory of the blue curve, and the blue trajectory could approach the predetermined flight trajectory.

In order to analyze PBVS better, we took the UAV as the origin to establish the coordinates, taking the longitude as the X-axis and the latitude as the Y-axis. From Figure 14b,c, we can see that the PBVS system couldaccurately reflect the position of UAV in the world coordinate system in practice. The actual flight speed can be obtained from Figure 14d. From Figure 14e, we can see that the error of PBVS was not more than 2 m. From Table 2, we can get the specific data of the box diagram in the simulation and actual environment.

Through the simulation and the actual flight test, it can be seen that PBVS could provide emergency navigation information in the environment without GNSS, so that the UAV could complete some tasks such as photographs. Compared with GPS, PBVS could provide a short-distance positioning signal free from communication signal interference, and its ability of positioning was also not weaker than GPS. To compare the proposed solution to those mentioned in Section 1, Table 3 summarizes the results obtained, highlighting the main differences between them.

## 5. Conclusions

In this paper, we proposed a pan-tilt-based visual servoing (PBVS) method for obtaining the world coordinate information. We utilized the vision system carried by the UAV for target recognition and leverage the PTZ control system to adjust the servo to move the target to the center of the field of view. The relative position relationship between the UAV and the anchor point could be calculated through geometry after information about the height of the UAV and the angle of PTZ was collected. When the latitude and longitude coordinates of the anchor point were known, the current position of the UAV including latitude and longitude information could be calculated based on the position calculation in the world coordinate system. The innovation of this paper was that changes of the PTZ angle were utilized to calculate the relative position relationship between the UAV and the target and obtain the real-time world coordinates of the UAV during the target tracking process. With experimental verification, the system could accurately calculate the coordinates of the UAV based on the coordinates of the target object. In the environment where the UAV positioning information is not available, this paper provides a novel approach to achieving absolute positioning.

## Figures and Tables

**Table 1:** Experimental platform models and parameters.

Hardware	Parameter
Camera	GoPro 1920*1080HD, 117 g
Image Transmission Equipment	Frequency range 750 MHz, average delay 300 ms
Data Transmission Equipment	The communication distance is 1 km, the highest data transmission rate 3300 B/s, and the average delay 5–10 ms
PTZ Controller	Self-made (STM32F103,72 MHz)
	IMU:MPU6500
Camera Attitude Measurement Unit	Magnetometer:LSM303D
	Barometer:MS5611
Battery Type	LIPO/22.2 V/12000 mAh/30 C
Ground Station	Intel XEON E5-2678 V3/RTX2080TI
RTK	10 Hz
Wheelbase	680 mm
Motors’ Max. Current	30 A
Brushless Motors	X4110S 340 KV
Brushless ECS	40 A
PTZ	3 axis
Autopilot	Self-made( STM32F407,168 MHz)
Payload Capability	5.5 KG
Hover Time	13 min

**Table 2:** Box chart data in the simulation and actual environment.

Environment	Category	Mean Value	Median	75th Percentile	25th Percentile	Max	Min	Outliers	RMSE
	Offset X	0.0804	0.0864	0.1061	0.0604	0.1478	0.0009	N/a	0.0882
Simulate	Offset Y	0.074	0.0888	0.1019	0.0391	0.1268	0.0008	N/a	0.0831
	Error Radius	0.1103	0.1268	0.1502	0.0729	0.1789	0.0011	N/a	0.1211
	Offset Latitude	0.587	0.4718	0.9879	0.1499	1.8759	0.0111	N/a	0.7677
Actual	Offset Longitude	0.5457	0.3297	0.8841	0.1199	1.8781	0.01	2.3377	0.7577
	Error Radius	0.8744	0.8282	1.427	0.2428	2.3541	0.0149	N/a	1.0786

**Table 3:** The performance of the different schemes.

Source	Accuracy (m)	Maximum Altitude (m)	Flight Speed (m/s)	Outdoor
GPS-based	1–3	N/a	N/a	Yes
Ours/outdoor	0.87	15	2	Yes
Ours/simulated	0.11	18	2–3	No
Wubben et al. [33]	1.2	20	0.1	Yes
Kim et al. [34]	5	140	15	Yes
